# Supplementary material for: Linker histone variant H1.2 is a brake on white adipose tissue browning
Source: Nat Commun. 2023 Jul 6;14:3982. doi: 10.1038/s41467-023-39713-w (PMC10325996; doi:10.1038/s41467-023-39713-w)
Supplement: Supplementary file 1 — Supplementary Information [file 41467_2023_39713_MOESM1_ESM.pdf]

Nature Communications

Supplementary information

for

Linker histone variant H1.2 is a brake on white adipose tissue browning

Yangmian Yuan[1], Yu Fan[1], Yihao Zhou[1], Rong Qiu[1], Wei Kang[1], Yu Liu[1], Yuchen Chen[2], Chenyu Wang[1], Jiajian Shi[2], Chengyu Liu[3], Yangkai Li[4], Min Wu[1], Kun Huang[2], Yong Liu[1], Ling Zheng[1]*

[1]Hubei Key Laboratory of Cell Homeostasis, Frontier Science Center for Immunology and Metabolism, College of Life Sciences, Wuhan University, Wuhan 430072, China

[2]School of Pharmacy, Tongji Medical College and State Key Laboratory for Diagnosis and Treatment of Severe Zoonotic Infectious Diseases, Huazhong University of Science and Technology, Wuhan 430030, China

[3]Department of Transfusion Medicine, Wuhan Hospital of Traditional Chinese and Western Medicine, Tongji Medical College, Huazhong University of Science and Technology, Wuhan 430030, China

[4]Department of Thoracic Surgery, Tongji Hospital, Tongji Medical College, Huazhong University of Science and Technology, Wuhan 430030, China

* Corresponding author

Ling Zheng, Ph.D.
College of Life Sciences
Wuhan University
Wuhan 430072, China
E-mail: lzheng@whu.edu.cn

Supplementary information includes 2 Supplementary Tables and 14 Supplementary Figures.

Supplementary Table 1. Primers used in the present study.

Supplementary Table 2. Primary antibodies used in the present study.

Supplementary Fig. 1. The transcriptional levels of somatic histone H1 variants in fat tissues of mouse and human.

Supplementary Fig. 2. H1.2 is increased upon adrenaline stimulation in iWAT and BAT.

Supplementary Fig. 3. Construction and identification of adipocyte specific H1.2 knockout mice.

Supplementary Fig. 4. Normal adipocyte morphology in young H1.2AKO mouse under normal chow-feeding.

Supplementary Fig. 5. No alteration in transcriptional levels of other somatic H1 variants and Ucp1-independent thermogenic genes in H1.2AKO mice after cold exposure.

Supplementary Fig. 6. No alteration in BAT of WT and H1.2AKO mice after cold exposure.

Supplementary Fig. 7. Normal chow-fed mice with H1.2 overexpression in beige adipocytes show reduced energy expenditure.

Supplementary Fig. 8. Similar *Il10rα* expression level in BAT of WT and H1.2AKO mice.

Supplementary Fig. 9. H1.2 knockout does not affect brown adipocyte thermogenesis *in vitro*.

Supplementary Fig. 10. H1.2 binds to *Il10rα* promoter and promotes *Il10rα* transcription.

Supplementary Fig. 11. Identification of whole-genome DNA-binding sites for H1.2 by ChIP-seq.

Supplementary Fig. 12. AAV-Il10rα treatment does not affect iWAT weights or inflammation under cold exposure.

Supplementary Fig. 13. H1.2AKO mice show improved metabolic status under HFD feeding.

Supplementary Fig. 14. Overexpression of Il10rα abolishes the metabolic improvement in HFD-fed H1.2AKO mice.

Supplementary Table 1. Primers used in the present study

Gene	Forward	Reverse
**Primer sequences for qPCR**		
H1.0	CTGCAACCCTTTGCCCATTC	GGTCTGTTTGCTGTCCTTGC
H1.1	TCGTGCAGGCAGTTTCTTCT	AGGCTTTTCAGCCCCAGTTT
H1.2	AAGGCGGCTTCTGGCGAGGCCA	CAGCACCAGTCGCCTTCTTA
H1.3	GAAGGCAGCAAAGAGTCCAG	CAGGACGCACCACTCTACTTC
H1.4	GCCAAGGCGAAAACGGTAAA	TGGGCTTCTAAGCAGTTGGC
H1.5	TGTAGAGAAGTCTCCCGCCA	GCGCTCCTTAGAGGCAGAAA
Fasn	TCTGGGCCAACCTCATTGGT	GAAGCTGGGGGTCCATTGTG
Acly	GGCCAGAGAGCTGGGTTTGA	CCCGAGCACAGATGATGGTG
Acaca	CCAGCTGATCCTGCGAACCT	GAACATTCCCGCAAGCCATC
Cidea	AGGGACAGAAATGGACACCG	GGTGACTCTGGCTATTCCCG
Cidec	GCTGAAGGGGCAGAAGTGGA	GCGCTTGGCCTTGTAGCAGT
Pgc1α	GAAATCCGAGCGGAGCTGAA	GAATAGGGCTGCGTGCCATC
Ucp1	GGCAACAAGAGCTGACAGTAAAT	GGCCCTTGTAAACAACAAAATAC
Cox8b	GAACCATGAAGCCAACGACT	GCGAAGTTCACAGTGGTTCC
Dio2	AGTCAAGAAGGTGGCATTCGA	ACAGCTTCCTCCTAGATGCCT
Prdm16	CAGCACGGTGAAGCCATTC	GCGTGCATCCGCTTGTG
Plin1	AGTCAGCGACAGCTTCTTCC	GGAAAGGCCCTTGACGAGAA
Cpt1a	CCATGATGGACCCCACAACA	TGGTCAACCTCCATGGCTCA
Cpt1b	ATCTTGGTGGCATGGCTGGT	GGGACTGGTCGATTGCATCC
Acox1	GGGAGTGCTACGGGTTACATG	CCGATATCCCCAACAGTGATG
Pparα	AGAGGGCTGAGCGTAGGTAA	ATTGGGCCGGTTAAGACCAG
Acot1	AGTGCTGATTCAAGGGCTGG	TTCTCGCAGCTGGATTGAAC
Acsl1	AGGACTCGGCATGTGACAAA	ACACCGCAGCAGAATCAGAA
Acsl3	GGTTGCTGAGTGGATTGCCA	AGCTTCATGGTTGATGGTGTT
Acsl4	CTTCCTCTTAAGGCCGGGAC	TGCCATAGCGTTTTTAGATTTCTT
Il4	CCATATCCACGGATGCGACA	CGTTGCTGTGAGGACGTTTG
Il6	CCACGGCCTTCCCTACTTC	TTGGGAGTGGTATCCTCTGTGA
Il10	GCTATGCTGCCTGCTCTTACT	CCTGCTGATCCTCATGCCA
Il10rα	CCAGGATGTTGTCGCGTTTG	GCAGTTCTGTCCCGTATGCAA
Il10rβ	CTTCTGGTGCCAGCTCTAGG	AGCAGGTACCTCCCACTGTA
Cd22	GCCAAGCGTGTGAGACTTTT	CCATCATTTCCTCCCTGGCA
Cd45	GGCGCATCAGAAGGGGATAA	CCAGCAAAGAGCAACAGAACC
Cd3	AAGTAATGAGCTGGCTGCGT	CAGGATGCCCCAGAAAGTGT
Il1b	TGCCACCTTTTGACAGTGATG	GTGCTGCTGCGAGATTTGAA
Arg1	CTCCAAGCCAAAGTCCTTAGAG	GGAGCTGTCATTAGGGACATCA
Cd206	GTGGGGACCTGGCAAGTATC	CACTGGGGTTCCATCACTCC
Ccl7	AAGAAGGGCATGGAAGTCTG	TCAAGGCTTTGGAGTTGGG
Ccl9	GCCCAGATCACACATGCAAC	AGGACAGGCAGCAATCTGAA
Cxcl13	ATTCAAGTTACGCCCCCTG	TTGGCACGAGGATTCACAC

Cxcl17	TGTGATCACGTCAAGGGCAG	CTGGAGGGTCTTTGCGACTT
Cebpa	CCTGCTGATCCTCATGCCA	CCCCAGCCGTTAGTGAAGAG
Cebpb	TTGATGCAATCCGGATCAAACG	CAGTTACACGTGTGTTGCGTC
Fabp4	GTCACCATCCGGTCAGAGAG	TCGACTTTCCATCCCACTTC
Pparγ	ACCCAGAGCATGGTGCCTTC	CCATCACGGAGAGGTCCACA
Adipoq	GCACTGGCAAGTTCTACTGCAAA	GTAGGTGAAGAGAACGGCCTT
Gatm	CTCCAACACCAGTCATCCCC	TCTACCATCACGCGCTTCTC
Gamt	ACTCCCCCTACTATGCCTTC	ACACTGAGGGACAGTGGAGA
Ckmt1	TCAGCCTTCATCGTGACCC	CTGGCGCAAGTCAGGAACTA
Serca2b	TTTGTGGCCCGAAACTACCT	TAATGAGCAGCACAAACGGC
Rn18s	CACCATCATGCAGAACCCACGAC	AGCCTCTCCAGGTCCTCACGC

Primer sequences for ChIP

mIl10rα P1	AACGCAGCGCTCAAGTCTCA	AGATTTTAACAGTTTCTGGGA
mIl10rα P2	CCCAGTCCTCACAGACAAGC	GTGGCCTAGACAGAGGATGC
mIl10rα P3	AGGCAGTAGTGTCTTCCGTG	GTCACTAGGATGCCCTTGCT
mIl10rα P4	CTCAAGGCGCCTATTGACAC	CCTGACTCTTGGTGGGAGTC
mGapdh P1	GGAGTGAAGAATCCCGGTCT	GGTGAGATCAGTGAGGGGAG
mGapdh P2	TGAGTCACTTGGAGCAGGAG	CTACCCAAAGAGCCCTTCCA
mGapdh P3	TGCACCCCAGAAAACATTCG	GTCATTTGCCAGTCTTCGGG
mGapdh P4	TGTTTGTGTGTGTGTGTGCA	GGTGGTTCTCTTGGGTGTCT
mNrp2 P1	CCAGTCATCTCTCTGCCCTT	ATGAAGGAAGAGGGGAAGCC
mNrp2 P2	GCCTCTAACTCCAGCCCTTT	TGGGTTCTTCTGGGTGGTTT
mNrp2 P3	TGTTGGAAGCTTGAAGGTGT	TGCGGTCTGGGTTAGGATTT
mNrp2 P4	CAGGTGGCATTGTGTTGGAT	TCAAAGTTAAGGTGCCACGC

Primer sequences for genotyping

Flox	TGAGACTTCTCGGATCCTTTACC	GCAGCAGGAGCAGCCTCAGACAT
Adi-cre	ACGGACAGAAGCATTTTCCA	GGATGTGCCATGTGAGTCTG

Sequences of shRNA

H1.2 #1	5'- TCTTAGCGCTCTTCTTCGG -3'
H1.2 #2	5'- GAAGCCAAGCCCAAGGTTA -3'

Supplementary Table 2. Primary antibodies used in the present study.

Antigen	Vendor	Catalog number	Dilution
H1.2 (IHC/IF/ChIP)	Abcam	ab181973	1:500 for IHC/IF;<br>2 μg/sample for ChIP
H1.2 (WB)	Abclonal	A0646	1:1000
Pparγ (WB)	Cell Signaling Technology	2435	1:1000
Ucp1 (WB/IHC)	Abcam	ab10983	1:1000–1:10000
Il10rα (WB/IF)	Abcam	ab225820	1:1000
Perilipin 1 (IF)	Abcam	ab61682	1:2000
Fabp4 (WB)	Cell Signaling Technology	2120	1:5000
α-Tubulin (WB)	Beyotime Biotechnology	AF0001	1:10000
Hsp70 (WB)	BD Pharmingen	610607	1:10000

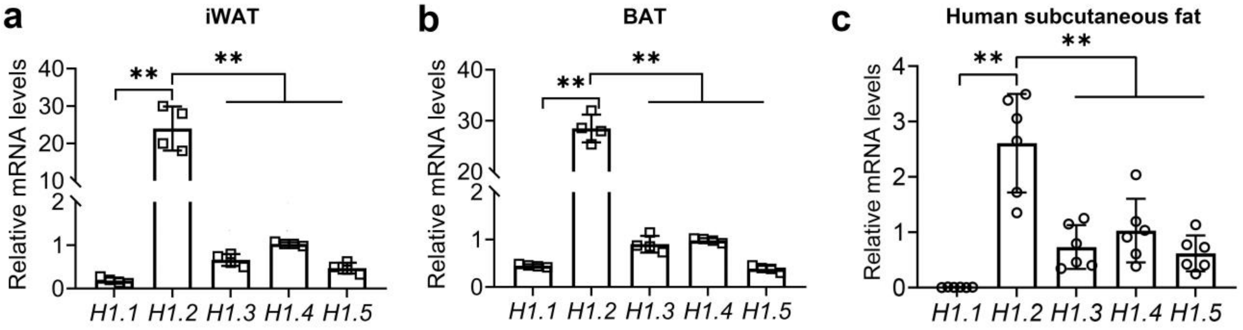

Supplementary Fig. 1. The transcriptional levels of somatic histone H1 variants in fat tissues of mouse and human. a Relative mRNA levels of histone *H1.1-H1.5* in iWAT of 10-week-old C57BL/6 male mice (n = 4). $P_{H1.2\text{ vs }H1.1} < 0.0001$, $P_{H1.2\text{ vs }H1.3} < 0.0001$, $P_{H1.2\text{ vs }H1.4} < 0.0001$, $P_{H1.2\text{ vs }H1.5} < 0.0001$. **b** Relative mRNA levels of histone *H1.1-H1.5* in BAT of 10-week-old C57BL/6 male mice (n = 4). $P_{H1.2\text{ vs }H1.1} < 0.0001$, $P_{H1.2\text{ vs }H1.3} < 0.0001$, $P_{H1.2\text{ vs }H1.4} < 0.0001$, $P_{H1.2\text{ vs }H1.5} < 0.0001$.**c** Relative mRNA levels of histone *H1.1-H1.5* in human subcutaneous fat (n = 6 for normal body mass index (BMI) subjects). $P_{H1.2\text{ vs }H1.1} < 0.0001$, $P_{H1.2\text{ vs }H1.3} < 0.0001$, $P_{H1.2\text{ vs }H1.4} < 0.0001$, $P_{H1.2\text{ vs }H1.5} < 0.0001$. Data are mean ± S.D.. One-way ANOVA with Dunnett's multiple comparisons test in **a**-**c**. $^{**}P < 0.01$. Source data are provided in a Source data file.

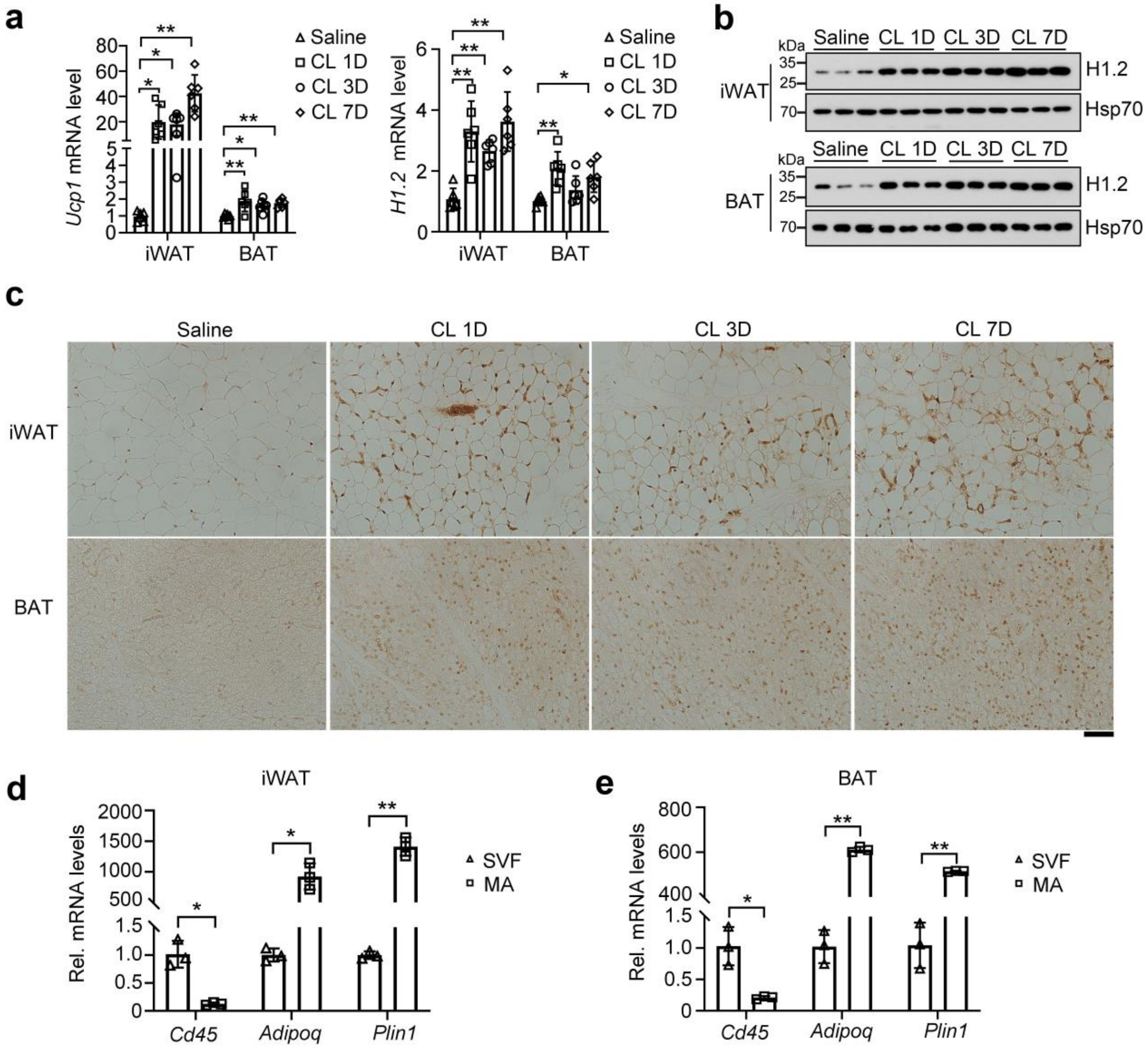

Supplementary Fig. 2. H1.2 is increased upon adrenaline stimulation in iWAT and BAT. 10-week-old male mice were used in this figure. **a** *Ucp1* and *H1.2* mRNA levels in iWAT and BAT of mice with CL316,243 (1 mg/kg, CL) treatment for 1 day (1D), 3 days (3D) and 7 days (7D) (n = 6 per group; one-way ANOVA with Tukey's multiple comparisons test). $P_{Ucp1\text{-iWAT (Saline vs CL 1D)}}$ = 0.0189, $P_{Ucp1\text{-iWAT (Saline vs CL 3D)}}$ = 0.0362, $P_{Ucp1\text{-iWAT (Saline vs CL 7D)}}$ < 0.0001, $P_{Ucp1\text{-BAT (Saline vs CL 1D)}}$ = 0.0016, $P_{Ucp1\text{-BAT (Saline vs CL 3D)}}$ = 0.0221, $P_{Ucp1\text{-BAT (Saline vs CL 7D)}}$ = 0.0052, $P_{H1.2\text{-iWAT (Saline vs CL 1D)}}$ = 0.0001, $P_{H1.2\text{-iWAT (Saline vs CL 3D)}}$ = 0.004, $P_{H1.2\text{-iWAT (Saline vs CL 7D)}}$ < 0.0001, $P_{H1.2\text{-BAT (Saline vs CL 1D)}}$ = 0.0014, $P_{H1.2\text{-BAT (Saline vs CL 3D)}}$ = 0.3855, $P_{Ucp1\text{-BAT (Saline vs CL 7D)}}$ = 0.0154. **b-c** Western blot analysis (b) and representative

immuno-histochemical staining (c) of H1.2 protein level in iWAT and BAT of mice with CL316,243 treatment for indicated time (n = 3 per group). Scale bar = 50 μm. **d-e** Relative mRNA levels of markers of stromal vascular fraction (SVF) cells (*Cd45*) and mature adipocytes (MA; *Adipoq* and *Plin1*) in iWAT (d) and BAT (e) of WT mice (n = 3 per group; unpaired two-tailed Student's t-test). Data are mean ± S.D.. *P < 0.05, **P < 0.01. Source data and exact P values are provided in a Source data file.

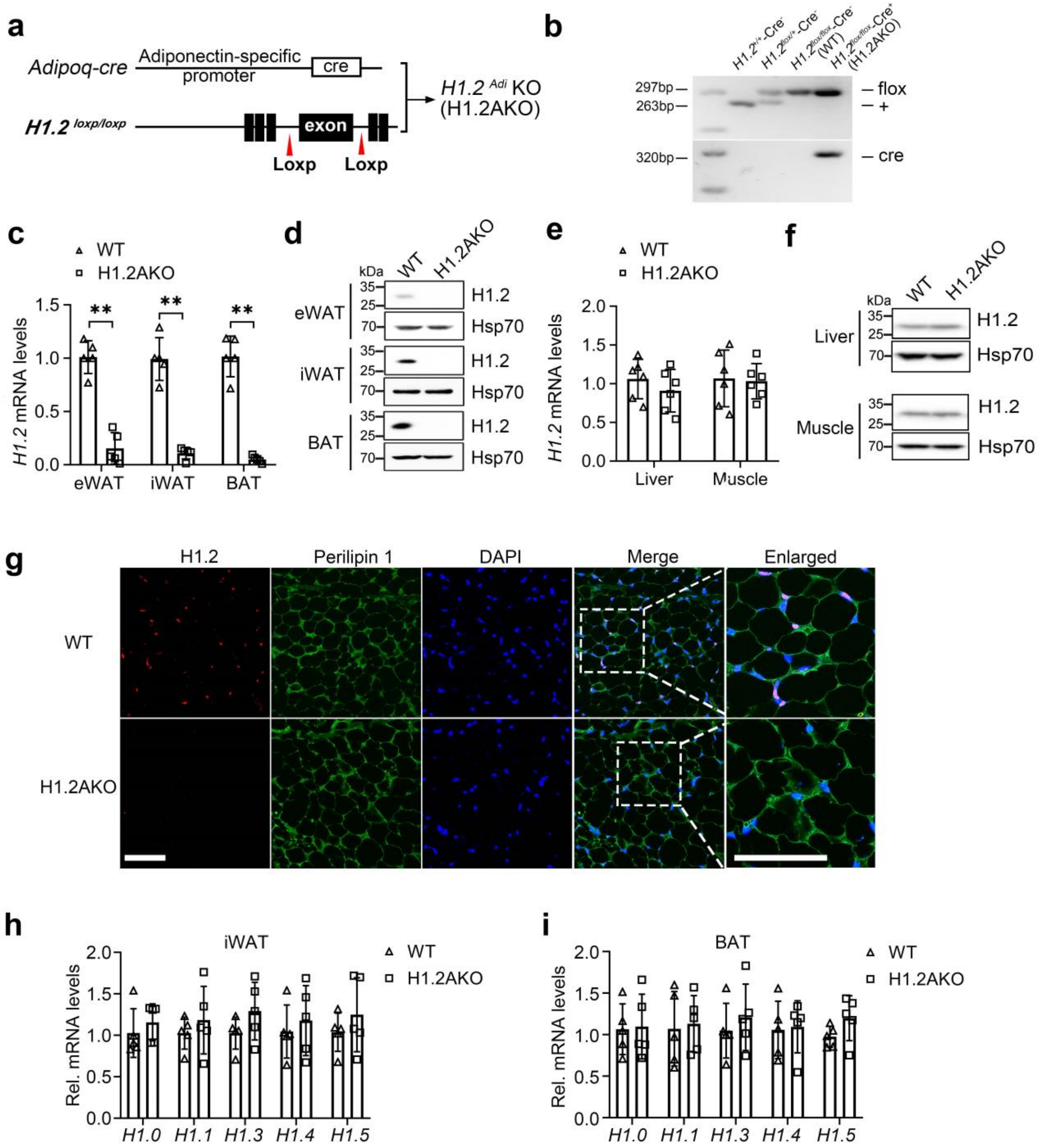

Supplementary Fig. 3. Construction and identification of adipocyte specific H1.2 knockout mice. 10-week-old male mice were used in this figure. **a** Schematic of the generation of adipocyte specific H1.2 knockout (H1.2AKO) mice. **b** Representative genotyping results of at least three independent experiments. **c** *H1.2* levels in eWAT/iWAT/BAT of WT and H1.2AKO mice (n = 5 per group; unpaired two-tailed Student's t-test). **d** Representative Western blots of H1.2 level in adipose tissues of WT and

H1.2AKO mice (pooled samples from two individual mice for each lane; repeated three independent experiments). **e** *H1.2* levels in the liver and muscle of WT and H1.2AKO mice (n = 6 per group; unpaired two-tailed Student's t-test). **f** Representative Western blots of H1.2 level in the liver and muscle of WT and H1.2AKO mice (pooled samples from two individual mice for each lane; repeated three times independently with similar results obtained). **g** Representative immunofluorescent results of H1.2 level in iWAT of WT and H1.2AKO mice (n = 3 per group). Scale bar = 50 μm. **h-i** Relative mRNA levels of other somatic H1 variants in iWAT (h) and BAT (i) of WT and H1.2AKO mice (n = 5 per group; unpaired two-tailed Student's t-test). Data are mean ± S.D.. $^{**}P < 0.01$, Source data and exact *P* values are provided in a Source data file.

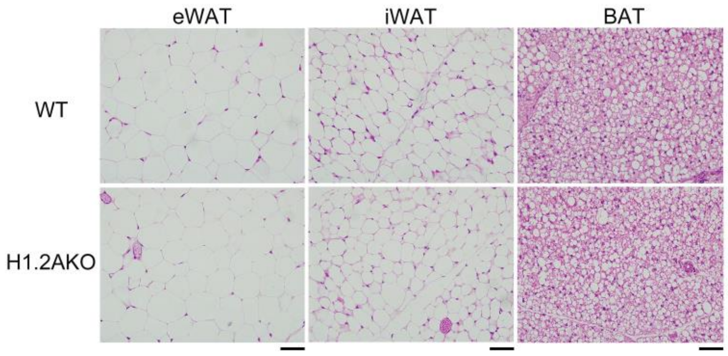

Supplementary Fig. 4. Normal adipocyte morphology in young H1.2AKO mouse under normal chow-feeding. Representative H&E staining of eWAT/iWAT/BAT of normal chow-fed WT and H1.2AKO male mice at 10-week-old (n = 5 per group). Scale bar = 50 μm.

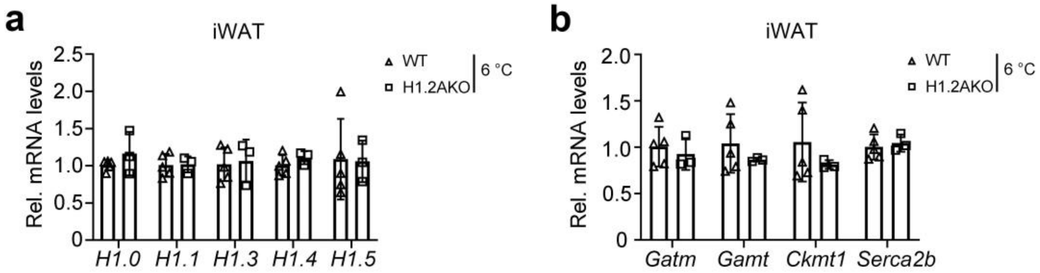

Supplementary Fig. 5. No alteration in transcriptional levels of other somatic H1 variants and Ucp1-independent thermogenic genes in H1.2AKO mice after cold exposure. 10-week-old male mice were used in this figure. **a** Relative mRNA levels of other somatic H1 variants in iWAT of WT and H1.2AKO mice after cold exposure (6 ℃ for 3 days; WT mice, n = 5; H1.2AKO mice, n = 3; unpaired two-tailed Student's t-test). **b** qPCR of Ca^{2+} and creatine cycling related genes of WT and H1.2AKO mice after cold exposure (6 ℃ for 3 days; WT mice, n = 5; H1.2AKO mice, n = 3; unpaired two-tailed Student's t-test). Data are mean ± S.D.. Source data and exact *P* values are provided in a Source data file.

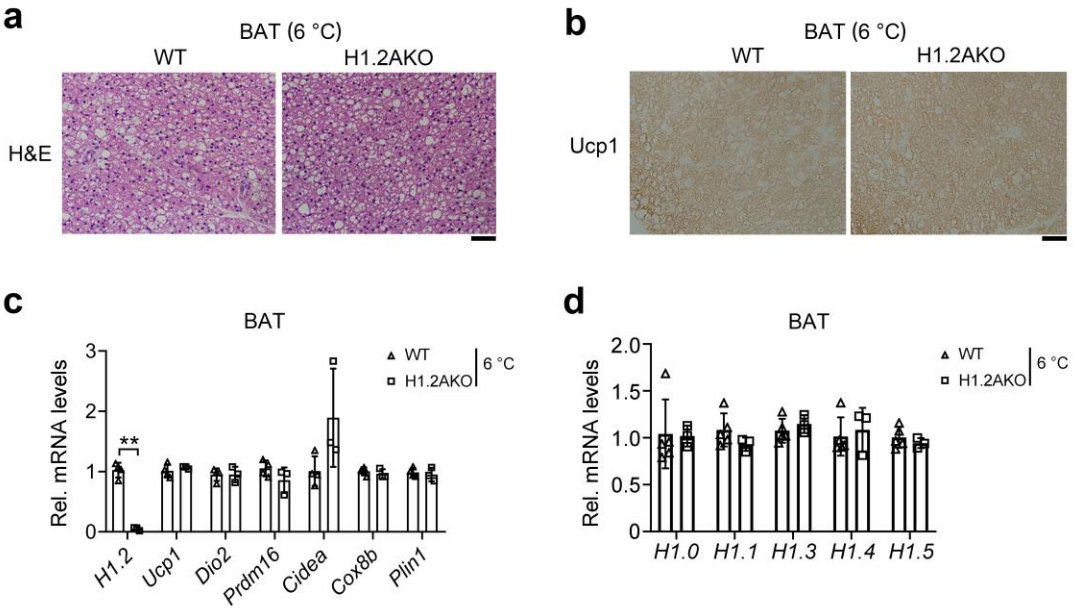

Supplementary Fig. 6. No alteration in BAT of WT and H1.2AKO mice after cold exposure. 10-week-old male mice were used in this figure. **a-b** Representative H&E (a) and Ucp1 staining (b) in BAT of WT and H1.2AKO mice after cold exposure at 6 ℃ for 3 days (WT mice, n = 4; H1.2AKO mice, n = 3). Scale bar = 50 μm. **c** *H1.2* and thermogenic gene levels in BAT of H1.2AKO mice after cold exposure (WT mice, n = 4; H1.2AKO mice, n = 3; unpaired two-tailed Student's t-test). **d** Relative mRNA levels of other somatic H1 variants in BAT of WT and H1.2AKO mice after cold exposure (WT mice, n = 5; H1.2AKO mice, n = 3; unpaired two-tailed Student's t-test). Data are mean ± S.D.. $^{**}P < 0.01$. Source data and exact *P* values are provided in a Source data file.

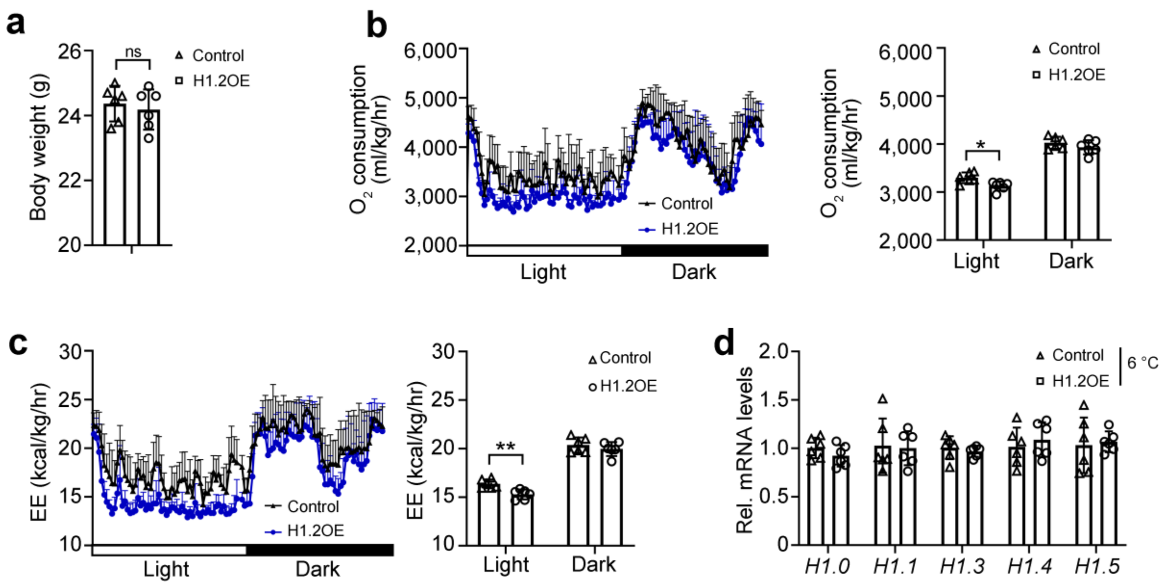

Supplementary Fig. 7. Normal chow-fed mice with H1.2 overexpression in beige adipocytes show reduced energy expenditure. 10-week-old male mice were used in this figure. **a** Body weight of C57BL/6 mice at 3 weeks after injected with AAV-Vehicle (Control) or AAV-H1.2 (H1.2OE) (n = 6; unpaired two-tailed Student's t-test). **b** Oxygen consumption with quantitative results of control and H1.2OE mice (n = 6 per group; two-tailed ANCOVA with body weight as a covariate). **c** energy expenditure (EE) with quantitative results of control and H1.2OE mice (n = 6 per group; two-tailed ANCOVA with body weight as a covariate). **d** Relative mRNA levels of other somatic H1 variants in iWAT of control and H1.2OE mice under cold exposure (6 °C for 3 days; n = 6 per group; unpaired two-tailed Student's t-test). Data are mean ± S.D.. **P* < 0.05, ***P* < 0.01; ns, not significant. Source data and exact *P* values are provided in a Source data file.

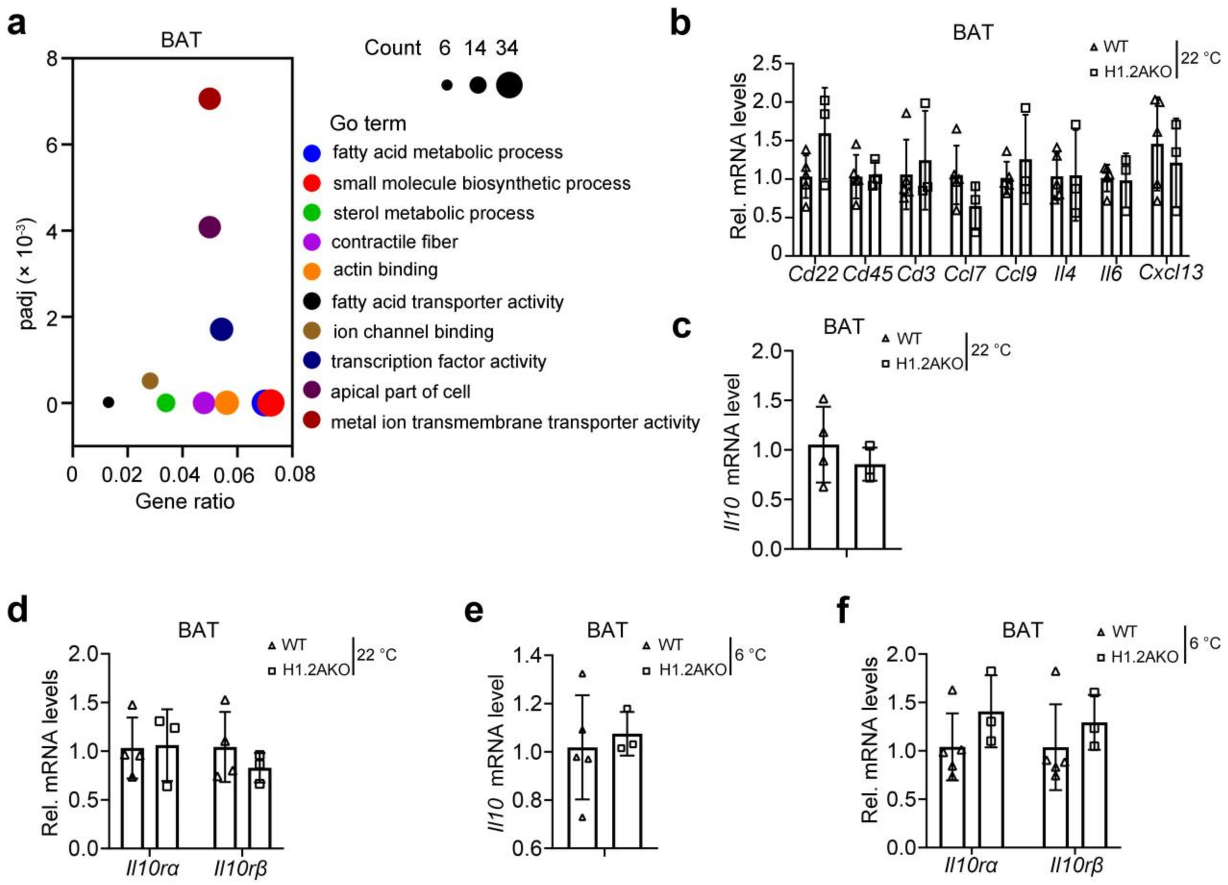

Supplementary Fig. 8. Similar Il10rα expression level in BAT of WT and H1.2AKO mice. 10-week-old male mice were used in this figure. **a** GO (Gene Ontology) analysis of differential expressed genes in BAT of WT and H1.2AKO mice. **b** mRNA levels of genes related to immune response in BAT of WT and H1.2AKO mice (WT mice, n = 5; H1.2AKO mice, n = 3; unpaired two-tailed Student's t-test). **c-d** *Il10* (c), *Il10rα* and *Il10rβ* (d) mRNA levels in BAT of WT and H1.2AKO mice under normal conditions (WT mice, n = 4; H1.2AKO mice, n = 3; unpaired two-tailed Student's t-test). **e-f** *Il10* (e), *Il10rα* and *Il10rβ* (f) mRNA levels in BAT of WT and H1.2AKO mice under cold stimuli (6 °C for 3 days) (WT mice, n = 5; H1.2AKO mice, n = 3; unpaired two-tailed Student's t-test). Data are mean ± S.D.. Source data and exact *P* values are provided in a Source data file.

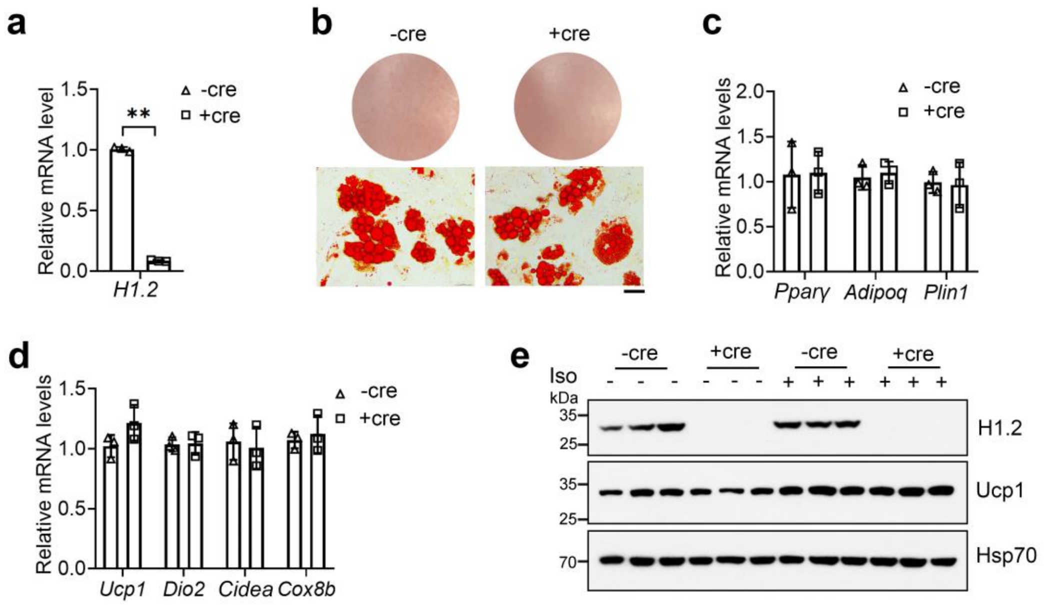

Supplementary Fig. 9. H1.2 knockout does not affect brown adipocyte thermogenesis *in vitro*. a *H1.2* level of differentiated brown adipocytes isolated from *H1.2*$^{flox/flox}$ mice treated with AAV-packed Cre recombinase (+Cre) or vehicle (-Cre) (n = 3; unpaired two-tailed Student's t-test; repeated three times independently with similar results obtained). **b** Representative Oil red O staining of differentiated brown adipocytes isolated from *H1.2*$^{flox/flox}$ mice treated with or without Cre (repeated three times independently with similar results obtained). Scale bar = 50 μm. **c-d** qPCR of genes related to adipogenesis (c) or thermogenesis (d) of differentiated brown adipocytes isolated from *H1.2*$^{flox/flox}$ mice treated with or without Cre (n = 3; unpaired two-tailed Student's t-test; repeated three times independently with similar results obtained). **e** H1.2 and Ucp1 protein levels of differentiated brown adipocytes isolated from *H1.2*$^{flox/flox}$ mice treated with or without Cre (n = 3; repeated three times independently with similar results obtained). Data are mean ± S.D.. $^{**}P < 0.01$. Source data and exact *P* values are provided in a Source data file.

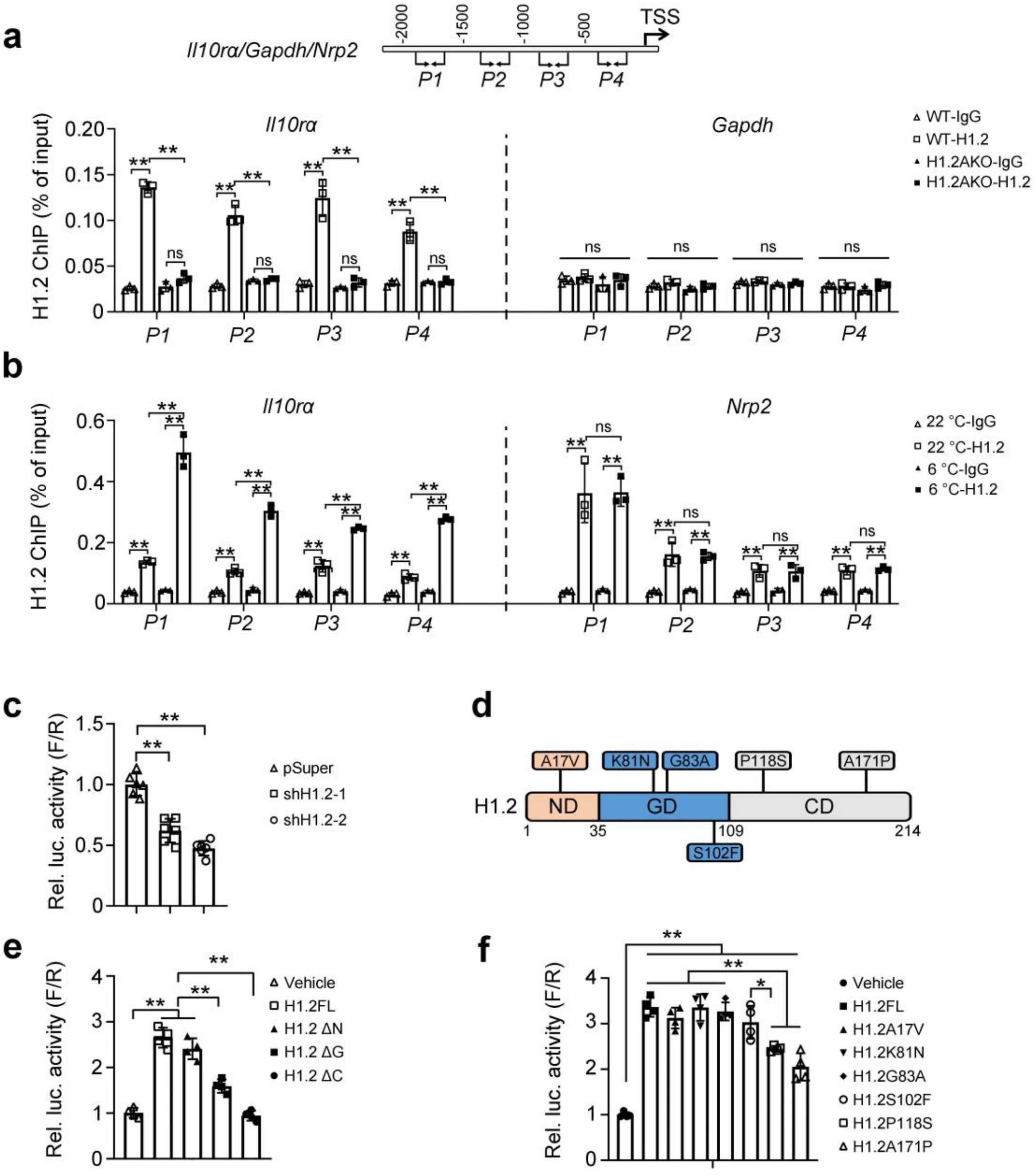

Supplementary Fig. 10. H1.2 binds to *Il10rα* promoter and promotes *Il10rα* transcription. a ChIP assay of H1.2 binding on different regions of the *Il10rα* promoter in iWAT of WT and H1.2AKO mice. Regions of *Gapdh* promoter are used as negative control (n = 3). $P_{Il10ra\text{-}P1/P2/P3/P4}$ (WT-IgG vs WT-H1.2) < 0.0001, $P_{Il10ra\text{-}P1/P2/P3/P4}$ (WT-H1.2 vs H1.2AKO-H1.2) < 0.0001, $P_{Il10ra\text{-}P1}$ (H1.2AKO-IgG vs H1.2AKO-H1.2) = 0.1967, $P_{Il10ra\text{-}P2}$ (H1.2AKO-IgG vs H1.2AKO-H1.2) = 0.9966, $P_{Il10ra\text{-}P3}$ (H1.2AKO-IgG vs H1.2AKO-H1.2) = 0.905, $P_{Il10ra\text{-}P4}$ (H1.2AKO-IgG

$_{\text{vs H1.2AKO-H1.2)}}$ > 0.999. **b** ChIP assay of H1.2 binding on different regions of the *Il10rα* promoter in iWAT of WT mice under normal conditions or cold stimuli (6 ℃ for 3 days). Regions of *Nrp2* promoter are used as negative control (n = 3). $P_{\textit{Il10ra-P1}\text{ (22 ℃-IgG vs 22 ℃-H1.2)}}$ = 0.0095, $P_{\textit{Il10ra-P2}\text{ (22 ℃-IgG vs 22 ℃-H1.2)}}$ = 0.0005, $P_{\textit{Il10ra-P3/P4}\text{ (22 ℃-IgG vs 22 ℃-H1.2)}}$ < 0.0001, $P_{\textit{Il10ra-P1/P2/P3/P4}\text{ (6 ℃-IgG vs 6 ℃-H1.2)}}$ < 0.0001, $P_{\textit{Il10ra-P1/P2/P3/P4}\text{ (22 ℃-H1.2 vs 6 ℃-H1.2)}}$ < 0.0001, $P_{\textit{Nrp2-P1}\text{ (22 ℃-IgG vs 22 ℃-H1.2)}}$ = 0.0003, $P_{\textit{Nrp2-P2}\text{ (22 ℃-IgG vs 22 ℃-H1.2)}}$ = 0.0004, $P_{\textit{Nrp2-P3}\text{ (22 ℃-IgG vs 22 ℃-H1.2)}}$ = 0.0026, $P_{\textit{Nrp2-P4}\text{ (22 ℃-IgG vs 22 ℃-H1.2)}}$ < 0.0001, $P_{\textit{Nrp2-P1}\text{ (6 ℃-IgG vs 6 ℃-H1.2)}}$ = 0.0004, $P_{\textit{Nrp2-P2}\text{ (6 ℃-IgG vs 6 ℃-H1.2)}}$ = 0.001, $P_{\textit{Nrp2-P3}\text{ (6 ℃-IgG vs 6 ℃-H1.2)}}$ = 0.0054, $P_{\textit{Nrp2-P4}\text{ (6 ℃-IgG vs 6 ℃-H1.2)}}$ < 0.0001, $P_{\textit{Nrp2-P1}\text{ (22 ℃-H1.2 vs 6 ℃-H1.2)}}$ = 0.9999, $P_{\textit{Nrp2-P2}\text{ (22 ℃-H1.2 vs 6 ℃-H1.2)}}$ = 0.9805, $P_{\textit{Nrp2-P3}\text{ (22 ℃-H1.2 vs 6 ℃-H1.2)}}$ = 0.9991, $P_{\textit{Nrp2-P4}\text{ (22 ℃-H1.2 vs 6 ℃-H1.2)}}$ = 0.8597. **c** Luciferase reporter assay of the effects of H1.2 knockdown on *Il10rα* level in HEK293T cells (n = 6 per group for each time; repeated three times independently with similar results obtained). $P_{\text{pSuper vs shH1.2-1}}$ < 0.0001, $P_{\text{pSuper vs shH1.2-2}}$ < 0.0001. **d** Structure and mutation sites of H1.2. **e-f** Luciferase reporter assay of the effects of H1.2 domain truncations (e) and different clinical H1.2 mutations (f) on *Il10rα* level in HEK293T cells (n = 4 per group for each time; repeated three times independently with similar results obtained). In **e**: $P_{\text{Vehicle vs H1.2FL}}$ < 0.0001, $P_{\text{Vehicle vs H1.2△N}}$ < 0.0001, $P_{\text{H1.2FL vs H1.2△G}}$ < 0.0001, $P_{\text{H1.2FL vs H1.2△C}}$ < 0.0001, $P_{\text{H1.2△N vs H1.2△G}}$ = 0.0006, $P_{\text{H1.2△N vs H1.2△C}}$ = 0.0005; in **f**: $P_{\text{Vehicle vs H1.2FL/H1.2A17V/H1.2K81N/H1.2G83A/H1.2S102F/H1.2P118S/H1.2A171P}}$ < 0.0001, $P_{\text{H1.2FL vs H1.2P118S}}$ = 0.0003, $P_{\text{H1.2A17V vs H1.2P118S}}$ = 0.01, $P_{\text{H1.2K81N vs H1.2P118S}}$ = 0.0003, $P_{\text{H1.2G83A vs H1.2P118S}}$ = 0.0013, $P_{\text{H1.2S102F vs H1.2P118S}}$ = 0.0351, $P_{\text{H1.2FL/H1.2A17V/H1.2K81N/H1.2G83A vs H1.2A171P}}$ < 0.0001. Data are mean ± S.D.. One-way ANOVA with Tukey's multiple comparisons test in **a-c** and **e-f**. *P < 0.05, **P < 0.01; ns, not significant. Source data and exact *P* values are provided in a Source data file.

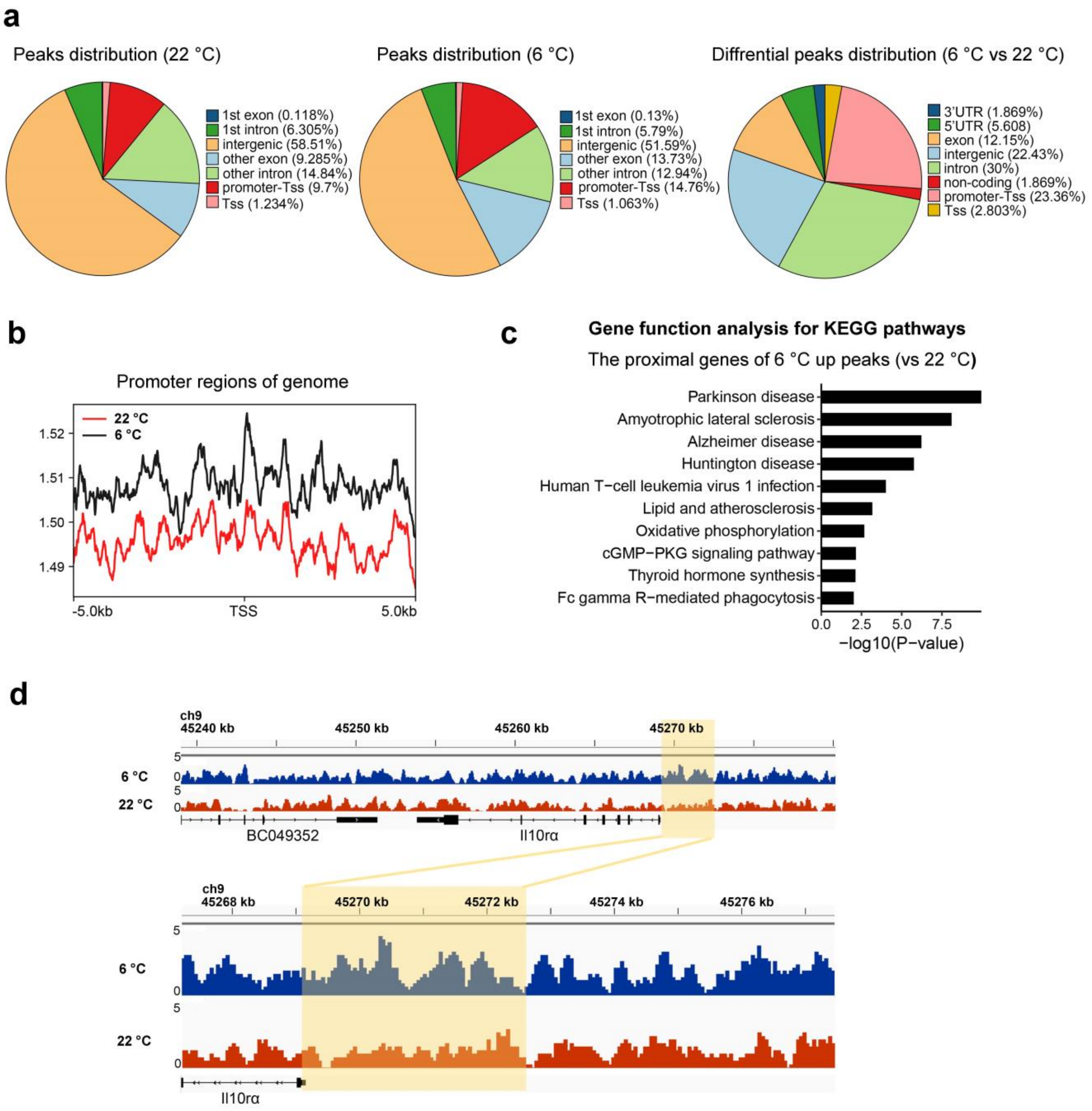

Supplementary Fig. 11. Identification of whole-genome DNA-binding sites for H1.2 by ChIP-seq. a Pie diagram showing the distribution of H1.2 binding sites in the genome including intergenic, 1st intron, 1st exon, promoter, transcription start site (TSS), other introns, and other exons in iWAT of WT mice under normal conditions, cold exposure (6 °C for 3 days), or differential binding sites under these two conditions. **b** The intensity of H1.2 peaks distributed on two sides of the TSS in iWAT of WT mice

under normal conditions and cold exposure. **c** KEGG pathway analysis of cold-induced differential peak-related genes enriched with H1.2. **d** ChIP-seq peaks of H1.2 enrichments at the promoter region of *Il10rα* under normal conditions and cold exposure.

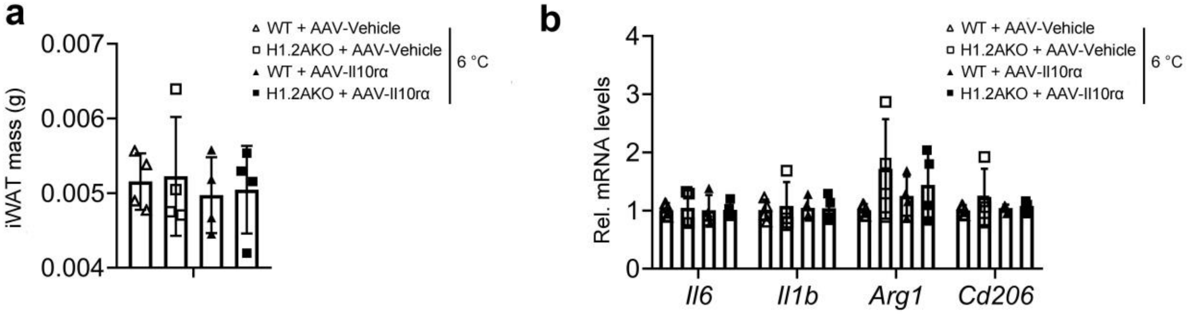

Supplementary Fig. 12. AAV-Il10rα treatment does not affect iWAT weights or inflammation under cold exposure. a Weights of iWAT of WT and H1.2AKO mice injected with AAV-Il10rα or AAV-Vehicle under cold exposure for 3 days (n = 4 per group; one-way ANOVA with Tukey's test). **b** mRNA levels of inflammation related genes in iWAT of WT and H1.2AKO mice injected with AAV-Il10rα or AAV-Vehicle under cold exposure (n = 4 per group; one-way ANOVA with Tukey's test). Data are mean ± S.D.. Source data and exact *P* values are provided in a Source data file.

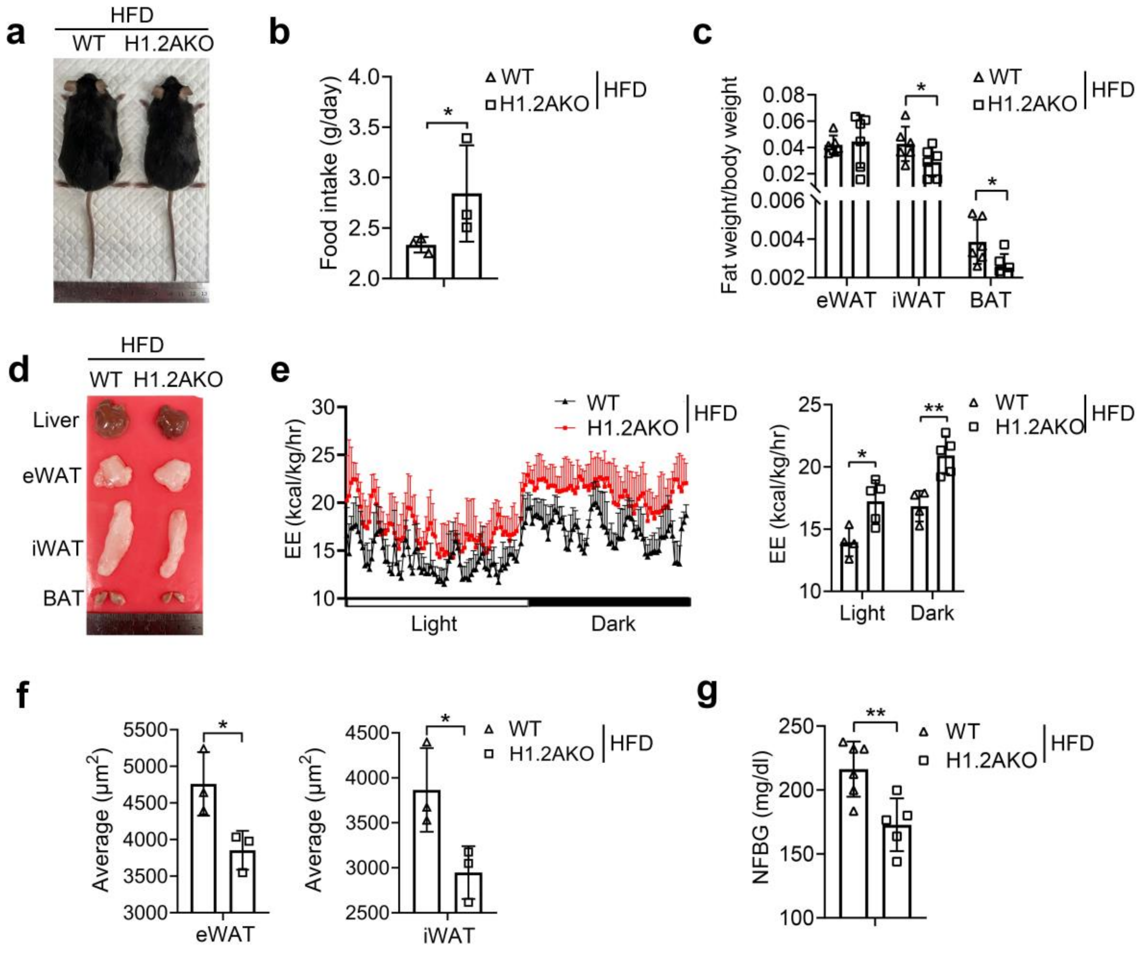

Supplementary Fig. 13. H1.2AKO mice show improved metabolic status under HFD feeding. 21-week HFD-fed WT and H1.2AKO male mice were used in this figure. **a** Representative view of HFD-fed WT and H1.2AKO mice (WT mice, n = 6; H1.2AKO mice, n = 5). **b** Food intake of HFD-fed WT and H1.2AKO mice (n = 3; unpaired two-tailed Student's t-test). **c-d** eWAT/iWAT/BAT mass (c) and abdominal view (d) of HFD-fed WT and H1.2AKO mice (WT mice, n = 6; H1.2AKO mice, n = 5; unpaired two-tailed Student's t-test). **e** Energy expenditure (EE) of HFD-fed WT and H1.2AKO mice (WT mice, n = 4; H1.2AKO mice, n = 5; two-tailed ANCOVA with body weight as a covariate). **f** Average adipocyte area of eWAT and iWAT in HFD-fed WT and H1.2AKO mice (n = 3; unpaired two-tailed Student's t-test). **g** Blood glucose of HFD-fed WT and H1.2AKO mice (WT mice, n = 6;

H1.2AKO mice, n = 5; unpaired two-tailed Student's t-test). Data are mean ± S.D.. *$P < 0.05$, **$P < 0.01$. Source data and exact P values are provided in a Source data file.

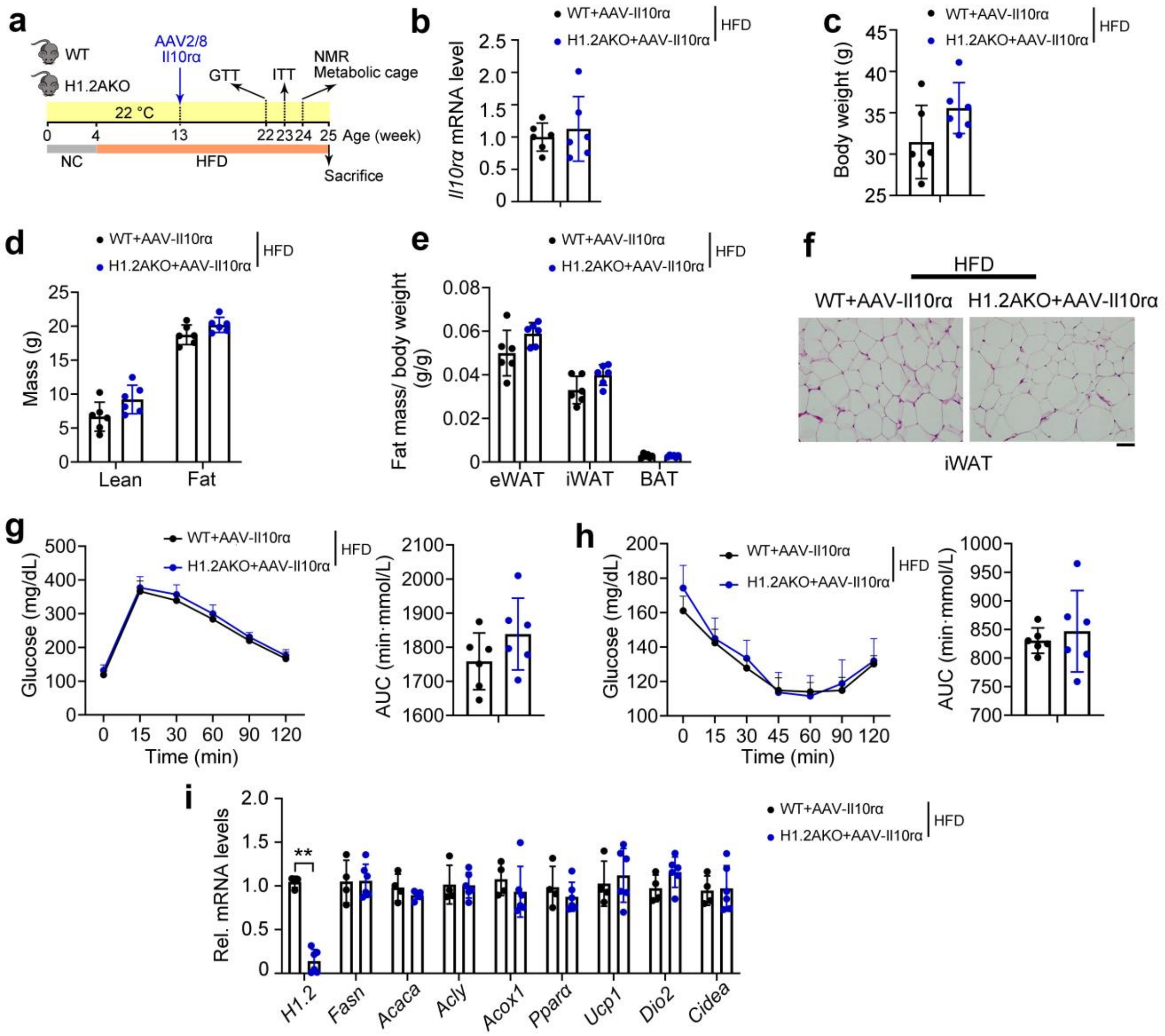

Supplementary Fig. 14. Overexpression of Il10rα abolishes the metabolic improvement in HFD-fed H1.2AKO mice. a Experimental design. HFD-fed WT and H1.2AKO mice were injected with AAV-Il10rα in both sides of iWAT at 13-week-old, and maintained on HFD-fed conditions until 25-week-old. **b** mRNA level of *Il10rα* in iWAT of AAV-Il10rα injected HFD-fed WT and H1.2AKO mice (n = 6 per group). **c-e** Body weight (c), fat mass (d), and tissue weights (e) of AAV-Il10rα injected HFD-fed WT and H1.2AKO mice (n = 6 per group). **f** Representative H&E staining of iWAT of WT and H1.2AKO mice (n = 6 per group). Scale bar = 50 μm. **g-h** GTT (g) and ITT (h) of AAV-Il10rα

injected HFD-fed WT and H1.2AKO mice (n = 6 per group). **i** qPCR of genes related to thermogenesis and lipid β-oxidation in iWAT of AAV- Il10rα injected HFD-fed WT and H1.2AKO mice (WT mice, n = 4; H1.2AKO mice, n = 6; unpaired two-tailed Student's t-test). Data are mean ± S.D.. $^{**}P < 0.01$. Source data and exact *P* values are provided in a Source data file.
