# Supplementary material for: Linker histone variant H1.2 is a brake on white adipose tissue browning
Source: Nat Commun. 2023 Jul 6;14:3982. doi: 10.1038/s41467-023-39713-w (PMC10325996; doi:10.1038/s41467-023-39713-w)
Supplement: Supplementary file 3 — Reporting Summary [file 41467_2023_39713_MOESM3_ESM.pdf]

nature portfolio

Corresponding author(s): Ling Zheng

Last updated by author(s): Jun 23, 2023

Reporting Summary

Nature Portfolio wishes to improve the reproducibility of the work that we publish. This form provides structure for consistency and transparency in reporting. For further information on Nature Portfolio policies, see our Editorial Policies and the Editorial Policy Checklist.

Statistics

For all statistical analyses, confirm that the following items are present in the figure legend, table legend, main text, or Methods section.

n/a	Confirmed	
☐	☒	The exact sample size (*n*) for each experimental group/condition, given as a discrete number and unit of measurement
☐	☒	A statement on whether measurements were taken from distinct samples or whether the same sample was measured repeatedly
☐	☒	The statistical test(s) used AND whether they are one- or two-sided<br>*Only common tests should be described solely by name; describe more complex techniques in the Methods section.*
☐	☒	A description of all covariates tested
☐	☒	A description of any assumptions or corrections, such as tests of normality and adjustment for multiple comparisons
☐	☒	A full description of the statistical parameters including central tendency (e.g. means) or other basic estimates (e.g. regression coefficient) AND variation (e.g. standard deviation) or associated estimates of uncertainty (e.g. confidence intervals)
☐	☒	For null hypothesis testing, the test statistic (e.g. *F*, *t*, *r*) with confidence intervals, effect sizes, degrees of freedom and *P* value noted<br>*Give P values as exact values whenever suitable.*
☒	☐	For Bayesian analysis, information on the choice of priors and Markov chain Monte Carlo settings
☒	☐	For hierarchical and complex designs, identification of the appropriate level for tests and full reporting of outcomes
☒	☐	Estimates of effect sizes (e.g. Cohen's *d*, Pearson's *r*), indicating how they were calculated

Our web collection on statistics for biologists contains articles on many of the points above.

Software and code

Policy information about availability of computer code

Data collection	Data collection for metabolic study: Oxymax (USA);<br>Data filtration for ChIP-seq: Trimmomatic version 0.36 (USA)
Data analysis	Image analysis for super-resolution immunofluorescence: Image J version 1.53 (USA);<br>analysis for western blots: Quantity One version 4.6.2 (USA)<br>Graphs: Graphpad Prism version 8 (USA);<br>Reads of ChIP-seq mapping to mouse genome : STAR version 2.5.3a (USA).;<br>Reads distribution analysis for ChIP-seq results: RSeQC version 2.6 (USA);<br>Peak calling for ChIP-seq results: MACS2 version 2.1.1 (USA) ;<br>Peaks annotation and peak distribution analysis for ChIP-seq results: The bedtools version 2.25.0 (USA);<br>Visualization of mapping results for DNA sequencing data: IGV version 2.16.1 (USA)

For manuscripts utilizing custom algorithms or software that are central to the research but not yet described in published literature, software must be made available to editors and reviewers. We strongly encourage code deposition in a community repository (e.g. GitHub). See the Nature Portfolio guidelines for submitting code & software for further information.

Data

Policy information about availability of data

All manuscripts must include a data availability statement. This statement should provide the following information, where applicable:

- Accession codes, unique identifiers, or web links for publicly available datasets
- A description of any restrictions on data availability
- For clinical datasets or third party data, please ensure that the statement adheres to our policy

RNA-seq data described in this work is available in NCBI GEO database under accession code GSE215412. ChIP-seq data generated in this study are accessible in NCBI GEO database through accession code GSE232530. Source data are provided with this paper.

Research involving human participants, their data, or biological material

Policy information about studies with human participants or human data. See also policy information about sex, gender (identity/presentation), and sexual orientation and race, ethnicity and racism.

Reporting on sex and gender	Sex and/or gender was not considered in the study design due to limited number of participants.
Reporting on race, ethnicity, or other socially relevant groupings	Asian (Chinese)
Population characteristics	40-70 years old subjects underwent surgery for benign or malignant lung tumor.
Recruitment	Human subcutaneous fat depots from both genders were obtained during surgery for lung tumor in Affiliated Tongji Hospital of Tongji Medical College (Wuhan, China). No self-selection bias was existed.
Ethics oversight	All performed procedures are approved by the ethics committee of Tongji Medical College in accordance with the principle of the Helsinki Declaration, and informed consent was obtained from all subjects.

Note that full information on the approval of the study protocol must also be provided in the manuscript.

Field-specific reporting

Please select the one below that is the best fit for your research. If you are not sure, read the appropriate sections before making your selection.

☒ Life sciences ☐ Behavioural & social sciences ☐ Ecological, evolutionary & environmental sciences

For a reference copy of the document with all sections, see nature.com/documents/nr-reporting-summary-flat.pdf

Life sciences study design

All studies must disclose on these points even when the disclosure is negative.

Sample size	Samples sizes were determined based on previous experience (doi: 10.1038/s41467-022-31476-0;doi: 10.2337/db20-0437;doi: 10.1002/mnfr.202100417), literature standards, or pilot data to ensure the possibility of statistical analysis and to minimize the use of experimental animals based on the 3R principles. Additional details regarding sample size are described in figure legends and can be found in Source Data.
Data exclusions	No data was excluded.
Replication	All experiments using animals were performed with at least three biological replicates; all experiments using cultured cells were independently performed for three times and similar results were obtained. Relevant information of replication was mentioned in indicated figure legends.
Randomization	Randomization was done to cell cultures. For animal studies, WT mice were randomly allocated to groups for cold, CL, or AAV treatment. Randomization was not done to other mice that were grouped based on genotypes (such as HFD vs. chow diet groups, or cold groups).
Blinding	Blinding was done for RNA-seq and ChIP-seq analysis. For the other animal studies and cell cultures, blinding was not done since the experimental design requires the investigators to know genotype information for each mouse or the treatment for cells. Most of the experiments were designed, performed and analyzed by the same person. However, no data was excluded in this study, all results were analyzed in unbiased ways.

Reporting for specific materials, systems and methods

We require information from authors about some types of materials, experimental systems and methods used in many studies. Here, indicate whether each material, system or method listed is relevant to your study. If you are not sure if a list item applies to your research, read the appropriate section before selecting a response.

Materials & experimental systems

n/a	Involved in the study
☐	☒ Antibodies
☐	☒ Eukaryotic cell lines
☒	☐ Palaeontology and archaeology
☐	☒ Animals and other organisms
☒	☐ Clinical data
☒	☐ Dual use research of concern
☒	☐ Plants

Methods

n/a	Involved in the study
☐	☒ ChIP-seq
☒	☐ Flow cytometry
☒	☐ MRI-based neuroimaging

Antibodies

Antibodies used

All antibodies used in this study were listed in Supplementary table 2.
Primary Antibodies:
Rabbit anti-H1.2 (ab181973, Abcam) EPR12690; dilution 1:500 for IHC/IF; 2 ug/sample for ChIP
Rabbit anti-H1.2 (A0646, Abclonal) ARC1836; dilution 1:1000 for WB;
Rabbit anti-Ucp1 (ab10983, Abcam); dilution 1:1000-10000 for WB/IHC;
Rabbit anti-PPARr (2435, Cell Signaling Technology) C26H12; dilution 1:1000 for WB;
Rabbit anti-Il10rα (ab225820, Abcam); dilution 1:1000 for WB/IF;
Goat anti-Perilipin 1 (ab61682, Abcam); dilution 1:2000 for IF;
Rabbit anti-Fabp4 (2120, Cell Signaling Technology) ; dilution 1:5000 for WB;
Rabbit anti-a-Tubulin (AF0001, Beyotime Biotechnology); dilution 1:10000 for WB;
Mouse anti-Hsp70 (610607, BD Pharmingen) dilution 1:10000 for WB.

Secondary antibodies:
Goat anti-rabbit IgG:HRP (Bio-Rad ,1706515); dilution 1:5000 for WB
Goat anti-mouse IgG:HRP (Bio-Rad,1706516); dilution 1:10000 for WB
Goat Anti-Rabbit IgG Antibody (H+L), Biotinylated (vector laboratories, BA-1000); dilution 1:1000 for IHC
Rabbit anti-Goat IgG (H+L) Alexa Fluor™ 488 (Thermo fisher, A11078); dilution 1:1000 for IF
Goat anti-Rabbit IgG (H+L) Alexa Fluor™ 594 (Thermo fisher, A11012); dilution 1:1000 for IF

Validation

These antibodies were selected carefully upon reviewing published papers and purchased from commercial vendors that have validated with over-expression and/or knockdown in cell lines. Their validation statements are available on the manufacturer's website.
Rabbit anti-H1.2 (ab181973, Abcam) EPR12690
validated for WB/IHC/IHC/ChIP in mouse and human cell lines
https://www.abcam.cn/products/primary-antibodies/histone-h12-antibody-epr12690-ab181973.html
Rabbit anti-H1.2 (A0646, Abclonal) ARC1836
validated for WB in mouse and human cell lines
https://abclonal.com.cn/catalog/A0646
Rabbit anti-Ucp1 (ab10983, Abcam)
validated for WB/IHC in mouse adipose tissue
https://www.abcam.cn/products/primary-antibodies/ucp1-antibody-ab10983.html
Rabbit anti-PPARr (2435, Cell Signaling Technology) C26H12
validated for WB in mouse cell lines
https://www.cellsignal.cn/products/primary-antibodies/pparg-c26h12-rabbit-mab/2435?site-search-type=Products&N=4294956287&Ntt=2435&fromPage=plp&_requestid=377958
Rabbit anti-Il10rα (ab225820, Abcam);
validated for WB in mouse and human cell lines
https://www.abcam.cn/products/primary-antibodies/il-10ra-antibody-ab225820.html
Goat anti-Perilipin 1 (ab61682, Abcam)
validated for WB /IHC/IF in mouse and human adipocytes
https://www.abcam.cn/products/primary-antibodies/perilipin-1-antibody-ab61682.html
Rabbit anti-Fabp4 (2120, Cell Signaling Technology)
validated for WB /IHC/IF in mouse adipocytes
https://www.cellsignal.cn/products/primary-antibodies/fabp4-antibody/2120?site-search-type=Products&N=4294956287&Ntt=2120&fromPage=plp&_requestid=380630
Rabbit anti-a-Tubulin (AF0001, Beyotime Biotechnology)
validated for WB /IHC/IF in mouse and human cell lines
https://www.beyotime.com/product/AF0001.htm
Mouse anti-Hsp70 (610607, BD Pharmingen)
validated for WB in mouse and human cell lines
https://www.bdbiosciences.com/zh-cn/products/reagents/microscopy-imaging-reagents/immunofluorescence-reagents/purified-mouse-anti-hsp70.610607

Eukaryotic cell lines

Policy information about cell lines and Sex and Gender in Research

Cell line source(s)	HEK293T was obtained from Procell Biotech (CL-0005, Wuhan, China)
Authentication	Authentication was done by Procell Biotech with morphology and STR profiling provided on https://www.procell.com.cn/view/471.html
Mycoplasma contamination	Cell lines were routinely monitored for mycoplasma contamination and negative results were obtained.
Commonly misidentified lines (See ICLAC register)	No commonly misidentified cell lines were used.

Animals and other research organisms

Policy information about studies involving animals; ARRIVE guidelines recommended for reporting animal research, and Sex and Gender in Research

Laboratory animals	LoxP-flanked H1.2 (H1.2flox/flox) mice, constructed by Saiye Inc. (Suzhou, China), and adiponectin-Cre mice (JAX, No.010803) were used to generate adipocyte-specific H1.2 knockout (H1.2AKO) mice. Male H1.2AKO and wildtype controls on C57BL/6 background ranging from 8-25 weeks age were used in this study with details of specific experiments mentioned in indicated figure legends and Methods section. 8-10 weeks wildtype C57BL/6 male mice were purchased from Hubei Provincial Center for Disease Control and Prevention. Unless noted otherwise, all mice were maintained in a specific-pathogen-free, temperature-controlled (22 °C ± 2 °C) animal facility with 50-60% humidity, a 12-h light/dark cycle, and free access to water and normal chow (#1025; HFK Bio., Beijing, China).
Wild animals	No wild animals were included in this study
Reporting on sex	Male mice of H1.2AKO and WT controls were used in this study; while female mice were used for breeding. This detail has also been described in Methods.
Field-collected samples	No field-collected samples included.
Ethics oversight	All performed procedures on animals have been approved by the Institutional Animal Care and Use Committee at the Institute of College of Life Science at Wuhan University

Note that full information on the approval of the study protocol must also be provided in the manuscript.

Plants

Seed stocks	*Report on the source of all seed stocks or other plant material used. If applicable, state the seed stock centre and catalogue number. If plant specimens were collected from the field, describe the collection location, date and sampling procedures.*
Novel plant genotypes	*Describe the methods by which all novel plant genotypes were produced. This includes those generated by transgenic approaches, gene editing, chemical/radiation-based mutagenesis and hybridization. For transgenic lines, describe the transformation method, the number of independent lines analyzed and the generation upon which experiments were performed. For gene-edited lines, describe the editor used, the endogenous sequence targeted for editing, the targeting guide RNA sequence (if applicable) and how the editor was applied.*
Authentication	*Describe any authentication procedures for each seed stock used or novel genotype generated. Describe any experiments used to assess the effect of a mutation and, where applicable, how potential secondary effects (e.g. second site T-DNA insertions, mosiacism, off-target gene editing) were examined.*

ChIP-seq

Data deposition

☒ Confirm that both raw and final processed data have been deposited in a public database such as GEO.

☒ Confirm that you have deposited or provided access to graph files (e.g. BED files) for the called peaks.

Data access links *May remain private before publication.*	https://www.ncbi.nlm.nih.gov/geo/query/acc.cgi?acc=GSE232530
Files in database submission	iWAT6_1.R1.fq.gz<br>iWAT22_1.R1.fq.gz<br>iWAT6_2.R1.fq.gz<br>iWAT6_1_IP.R1.fq.gz<br>iWAT22_2.R1.fq.gz

iWAT6_3.R1.fq.gz
iWAT6_2_IP.R1.fq.gz
iWAT22_1_IP.R1.fq.gz
iWAT6_3_IP.R1.fq.gz
iWAT22_2_IP.R1.fq.gz
iWAT6_1.R2.fq.gz
iWAT22_1.R2.fq.gz
iWAT6_2.R2.fq.gz
iWAT6_1_IP.R2.fq.gz
iWAT22_2.R2.fq.gz
iWAT6_3.R2.fq.gz
iWAT6_2_IP.R2.fq.gz
iWAT22_1_IP.R2.fq.gz
iWAT6_3_IP.R2.fq.gz
iWAT22_2_IP.R2.fq.gz
iWAT6_1.bw
iWAT22_1.bw
iWAT6_2.bw
iWAT6_1_IP.bw
iWAT22_2.bw
iWAT6_3.bw
iWAT6_2_IP.bw
iWAT22_1_IP.bw
iWAT6_3_IP.bw
iWAT22_2_IP.bw

Genome browser session
(e.g. UCSC)

All bigwig files have been deposited to GEO dataset as processed files.

Methodology

Replicates

For 22°C group, data are from biological duplicates; for 6 °C group, data are from biological triplicates.

Sequencing depth

Each library contains at least 90 million reads, with more than 90% of total reads are mapped and 80% of total reads are uniquely mapped. 200-500 bps, paired ended reads are enriched.

Antibodies

Rabbit anti-H1.2 (181973, Abcam)

Peak calling parameters

The MACS2 software（Version 2.1.1） was used for peak calling. epic --treatment {ip_beds} --control {input_beds} --number-cores {threads} --outfile {output} --bed {bbeedd} --chromsizes {genome_size} --false-discovery-rate-cutoff 0.00001 --effective-genome-fraction 1 --log {log_file}.

Data quality

FastQC (Version v0.11.5) was used for quality check of sequencing reads

Software

The RSeQC（version 2.6） was used for reads distribution analysis.The MACS2 software（Version 2.1.1） was used for peak calling. The bedtools（Version 2.25.0） was used for peaks annotation and peak distribution analysis.
